# Evaluation of the Needs for Palliative Care in Madeira Island: A Pre-pandemic Overview

**DOI:** 10.7759/cureus.34793

**Published:** 2023-02-09

**Authors:** Pedro Agrela, João Freitas, Sofia Rei, Ricardo Pinto, Sónia Freitas, Graciela Camacho, Yaneth Gonçalves, Marina Gouveia, Maria Gomes

**Affiliations:** 1 Family Medicine, Serviço de Saúde da Região Autónoma da Madeira, Entidade Pública Empresarial da Região Autónoma da Madeira (SESARAM, EPERAM), Funchal, PRT; 2 Family Medicine, Centro de Saúde do Bom Jesus, Funchal, PRT; 3 Family Medicine, Centro de Saúde do Caniço, Santa Cruz, PRT; 4 Family Medicine, Centro de Saúde Dr. Rui Adriano de Freitas, Funchal, PRT; 5 O Centro de Investigação Dra. Maria Isabel Mendonça, Serviço de Saúde da Região Autónoma da Madeira, Entidade Pública Empresarial da Região Autónoma da Madeira (SESARAM, EPERAM), Funchal, PRT; 6 Palliative Care Unit, Hospital Dr. João de Almada, Funchal, PRT

**Keywords:** multidisciplinary care, chronic disease, family medicine, needs evaluation, palliative care

## Abstract

Introduction

As general practitioners/family physicians, it is our duty to promote health and prevent disease by providing cure, care, or palliation. Palliative care (PC) plays a crucial role in integrated and patient-centered health services. In the Autonomous Region of Madeira (RAM), Portugal, the PC Unit (PCU) was established in 2012. According to data from 2010, 41.2% of individuals who passed away in this region had a requirement for PC. Our objective is to determine the potential needs for PC in our population in the year 2019, prior to the pandemic. Additionally, we aim to determine the main indications for PC and the number of emergency department admissions/hospitalizations and compare these needs with data from 2010.

Methods

We conducted an observational, cross-sectional, and descriptive, analytical study, in which the target population consisted of all individuals who passed away in RAM in the year 2019. From this population, a representative sample of deceased individuals was obtained. The gender, age, disease with a potential indication for palliative care, admissions to emergency care and hospitalizations in the preceding 12 months, and county of residence were obtained from the RAM Health Services and analyzed using Microsoft Excel (Microsoft Corp., Redmond, WA, USA) and the Statistical Package for the Social Sciences (SPSS) (IBM SPSS Statistics, Armonk, NY, USA) software.

Results

From the total number of individuals who passed away in RAM in the year 2019 (N=2,840), a representative sample of 339 deceased individuals was obtained. Of the deceased individuals, 56% presented with potential indications for palliative care, compared to the 41.2% result recorded in 2010 (p<0.0001). Among these individuals, 51.9% were female, and the average age was 79.7±12.2 years. Neoplasms were the primary indication for palliative care, accounting for 22.7%. Individuals with potential indications for PC, when compared to those without indications, had a higher number of hospitalizations and emergency episodes in the preceding 12 months (p=0.0005 and p=0.008, respectively).

Conclusion

We conclude that over half of the patients who passed away in RAM had potential indications for palliative care. These individuals experienced a higher number of hospitalizations and emergency episodes in the preceding 12 months. In this study, we also observed a significant increase in the need for palliative care compared to the year 2010.

## Introduction

The World Health Organization (WHO) defines palliative care (PC) as a method for enhancing the quality of life for patients and their families who are facing end-of-life situations [1]. The objective of this care approach is to alleviate suffering through early detection, evaluation, and treatment of physical, psychosocial, and spiritual pains and related issues [1].

In Portugal, the Palliative Care Law regulates the accessibility of PC and defines it as a proactive, integrated, and comprehensive care provided by specialized teams to both inpatients and outpatients, as well as their families, who are suffering from an incurable and progressive disease in its advanced stage [2]. This law aligns with the WHO’s goals and emphasizes the importance of early identification and effective treatment of physical, psychosocial, and spiritual pains and related issues as the foundation of this type of care [1,2].

The Palliative Care Network of the Autonomous Region of Madeira (Rede de Cuidados Paliativos da Região Autónoma da Madeira (RCP)) was founded in October 2012 and operates under the same organizational structure as the National Palliative Care Network (Rede Nacional de Cuidados Paliativos (RNCP)) [3]. The network is composed of a multidisciplinary team that includes a community-based team providing support to outpatients, an in-hospital team providing care to inpatients at Hospital Dr. João de Almada, and a consulting team supporting hospital services at Hospital Dr. Nélio Mendonça and Hospital dos Marmeleiros [4].

From 2010 to 2019, the resident population of the Autonomous Region of Madeira (RAM) increased from 247,568 to 254,254. In 2019, women made up a larger proportion of the population compared to men (53.3% and 46.7%, respectively), and the elderly represented 17% of the population, which was higher than in 2010 (13%). In 2019, the municipality of Funchal had the largest number of residents (104,024), followed by Santa Cruz (45,281) and Câmara de Lobos (33,675), while São Vicente and Porto Moniz had the smallest populations (5,143 and 2,342, respectively) [5]. In terms of mortality, there were 2,679 deaths in 2019, with a mortality rate of 10.5%. The majority of deaths were among women (52.1%). The municipality with the most deaths was Funchal (1,235), and the leading cause of death was cardiovascular diseases (797 deaths, with a mortality rate of 313.7 per 100,000 individuals), followed by Neoplasms (622 deaths, with a mortality rate of 244.8 per 100,000 individuals) [5,6].

The need for palliative care (PC) in RAM was initially evaluated by Camacho in 2012, with reference to the year 2010. In that year, 41.2% of individuals who died in RAM had potential indications for PC, with the most common indication being a diagnosis of a neoplasm (61.6%) [7].

The primary objective of this study is to assess the potential PC needs of patients who died in RAM in the year 2019. The secondary objectives of this study include identifying the main indications for PC, determining the number of emergency department admissions and hospitalizations in the preceding 12 months, and comparing these needs with data from 2010.

## Materials and methods

This study was conducted in compliance with the principles outlined in the Declaration of Helsinki and received approval from the Ethics Committee, and Scientific and Research Committee of Serviço de Saúde da Região Autónoma da Madeira, Entidade Pública Empresarial da Região Autónoma da Madeira (SESARAM, EPERAM).

This study was designed as an observational, cross-sectional, descriptive, analytical study with the target population being all individuals who passed away in RAM in the year 2019. A representative sample of the deceased population was obtained through stratified random sampling applied to the 11 municipal areas in RAM, with a 95% confidence interval. The stratified sample was determined based on sex, age, and county of residence, which were provided by the Statistics Department of SESARAM, EPERAM.

Data was collected by consulting the clinical records of the deceased individuals, which were available on the informatics system of SESARAM, EPERAM (ATRIUM), and the online platform of the Death Certificate Information System (Sistema de Informação dos Certificados de Óbito (SICO)). Only clinical records with sufficient information were included in the study. When a clinical record with insufficient information was found, the individual immediately following was chosen. The data collected included sex, age, county of residence, number of hospital admissions (emergency department and hospitalization) in the last 12 months of life, existence of a potential indication for PC, and place of death of the individuals.

Regarding the potential indications for PC, the methodology of McNamara et al. [8] and Camacho [7] was employed. This methodology involved the consideration of the following 10 potential indications: cancer, heart failure, renal failure, liver failure, chronic obstructive pulmonary disease, motor neuron disease/amyotrophic lateral sclerosis, Parkinson’s disease, Huntington’s disease, Alzheimer’s disease, and human immunodeficiency virus (HIV)/acquired immunodeficiency syndrome (AIDS).

In this study, univariate analysis was performed to describe categorical variables using absolute and relative frequencies. To analyze the relationship between qualitative variables, the chi-square test (χ2) and Fisher’s exact test were applied, while Student’s t-test was used for quantitative variables. A statistical significance level of p<0.05 was considered. Data processing and statistical analysis were performed using Microsoft Excel (Microsoft Corp., Redmond, WA, USA) and Statistical Package for the Social Sciences (SPSS) version 25 (IBM SPSS Statistics, Armonk, NY, USA).

These methods were a replication of the study of Camacho [7] and applied to the year 2019.

## Results

A random sample of 339 individuals who passed away between January 1st and December 31st of 2019 was selected from all residents who died in RAM (N=2,840). The sample is composed of 51.9% (n=179) female individuals and 48.1% (n=163) male individuals, and the average age was 79.7±12.2 years. There was no statistically significant difference in gender distribution between the studied sample and the sample from the 2010 study (p=0.872) [7]. Upon comparing the number of deceased individuals per county, no substantial differences were observed between 2010 and 2019 (p=0.996). Funchal remained the county with the highest number of deaths in 2010 and 2019, accounting for 44.8% and 47.2%, respectively.

The most prevalent pathologies with potential indications for palliative care in the studied population were neoplasms (22.7%), dementia (15.3%), and congestive heart failure (8.8%). In 2010, neoplasms (25.4%) and dementia (12.8%) also topped the list, with the only difference being the third place, where chronic respiratory failure was identified (1.2%). A significant increase in the number of cases of congestive heart failure, chronic liver failure, and renal failure was observed between the two evaluation periods (p<0.0001) (Table 1).

**Table 1:** Diseases with potential indications for PC in the studied population who died in the years 2010 and 2019 (p<0.0001). PC: palliative care

Potential indications for palliative care		Year	
		2010	2019
Neoplasm	Number	85	77
	%	25.4%	22.7%
Dementia	Number	43	52
	%	12.8%	15.3%
Chronic respiratory failure	Number	4	13
	%	1.2%	3.8%
Congestive heart failure	Number	2	30
	%	0.6%	8.8%
Chronic liver failure	Number	2	7
	%	0.6%	2.1%
Renal failure	Number	2	11
	%	0.6%	3.2%
Total	Number	138	190
	%	41.2%	56%

There was a significant increase in potential indications for palliative care, from 41.2% in 2010 to 56% in 2019 (p<0.0001) (Table 2). The majority of deceased individuals with potential indications for palliative care in the study sample were female (53.2%). Neoplasms remained the primary indications for palliative care in this group. Compared to 2010, a higher number of patients died of respiratory failure, congestive heart failure, chronic liver failure, and renal failure in 2019 (p<0.0001) (Table 3).

**Table 2:** Population with potential indications for PC in the years 2010 and 2019 (p<0.0001). PC: palliative care

Potential indications for palliative care		Year	
		2010	2019
No	Number	197	149
	%	58.8%	44%
Yes	Number	138	190
	%	41.2%	56%
Total	Number	335	339
	%	100%	100%

**Table 3:** Distribution of the diseases in the groups with potential indications for PC (p<0.0001). PC: palliative care

Potential indications for palliative care		Year	
		2010	2019
Neoplasm	Number	85	77
	%	61.60%	40.50%
Dementia	Number	43	52
	%	31.20%	27.40%
Chronic respiratory failure	Number	4	13
	%	2.90%	6.80%
Congestive heart failure	Number	2	30
	%	1.40%	15.80%
Chronic liver failure	Number	2	7
	%	1.40%	3.70%
Renal failure	Number	2	11
	%	1.40%	5.80%
Total	Number	138	190
	%	100%	100%

Furthermore, it was observed that the majority of the studied population died in a hospital setting. After controlling for the place of death through homogenization of the sample, there were statistically significant differences between the years 2010 and 2019 (p=0.014) (Table 4). This comparison was made by excluding 19 individuals who passed away in the Palliative Care Unit (PCU) and one individual who died outside the major categories of locations. We observed an increase in deaths in private residences, emergency services, and nursing homes in the year 2019. In contrast, deaths in the context of hospitalization decreased from 78.3% to 65.1%.

**Table 4:** Distribution of deceased individuals with potential indications for PC in the years 2010 and 2019 by place of death (p=0.014). PC: palliative care

Places of death		Year	
		2010	2019
Hospital	Number	108	111
	%	78.30%	65.29%
Residence	Number	12	27
	%	8.70%	15.88%
Emergency service	Number	13	19
	%	9.40%	11.18%
Nursing home	Number	5	13
	%	3.60%	7.65%
Total	Number	138	170
	%	100%	100%

Regarding the emergency episodes in the 12 months prior to death among patients with potential indications for PC, the mean value was found to be 3.384±2.594 episodes per patient. There were statistically significant differences when compared to patients without indications for PC (p=0.008). A statistically significant difference was also observed in the average number of hospitalizations in the 12 months preceding death in the same population (p=0.0005), with a mean value of 1.863±1.420 hospitalizations per individual with a potential indication for PC (Table 5).

**Table 5:** Hospitalizations, emergency episodes, and age distribution between individuals with and without potential indications for PC. PC: palliative care

Category	Potential indications for PC	Number	Mean value	Standard deviation	p-value
Hospitalization	No	149	1.329	1.326	0.0005
	Yes	190	1.863	1.420	
Emergency episodes	No	149	2.644	2.594	0.008
	Yes	190	3.384	2.491	
Age	No	149	76.550	14.343	0.873
	Yes	190	76.784	12.602	

## Discussion

A comparison between the two evaluation periods reveals a significant change in the number of cases that demonstrate potential indications for palliative care (PC) between 2010 and 2019. Although neoplasms continue to be the primary indication for PC, there was a decrease of 21.1% in such cases in 2019, a reduction of approximately 1.5 times. Similarly, the incidence of dementia as an indication for PC also saw a slight decrease, from 31.2% to 27.4%, a reduction of approximately 1.1 times.

The frequency of various diseases has statistically significantly increased, as evidenced by statistical analysis (p<0.0001). For instance, the incidence of chronic respiratory failure has more than doubled, rising from 2.9% to 6.8%. Similarly, congestive heart failure has increased dramatically, rising from 1.4% to 15.8% (which is approximately 11 times higher than in 2010). Chronic liver failure, which accounted for 1.4% of the potential indications for PC-related deaths in 2010, has increased to 3.7% in 2019, representing a 2.6-fold increase. Lastly, renal failure has seen an increase from 1.4% to 5.8%, which corresponds to a fourfold increase.

The observed variations in oncological pathology can be partially attributed to the significant advancements in cancer treatment over the past decade, which have led to increased survival rates among patients [9]. The rise in other causes can be explained by the aging of the population in RAM, with the proportion of elderly individuals increasing from 12% in 2010 to 17% in 2019 [5,7]. Additionally, advancements in the treatment of acute causes have allowed patients to progress to and eventually succumb to chronic illnesses.

According to the study of Camacho from 2010, approximately 41.2% of the deceased population in the RAM region had potential indications for PC [7]. In 2019, a marked increase was observed, rising to 56% (p<0.0001). This increase aligns with the values suggested by various authors, which typically range close to 60% in any population [10,11].

According to some studies, it has been established that the majority of patients would prefer to pass away at home if given the choice [12]. Hence, the percentage of deaths occurring at home is considered a measure of the quality of PC services [7,13]. Most individuals, however, die in a hospital setting, which can be viewed as an unfavorable outcome. After homogenizing the sample for the place of death, statistically significant differences were observed between patients who had indications for palliative care and those who did not (p=0.014).

Compared to 2010, we observed that patients with potential indications for palliative care passed away more frequently in their homes and less frequently in a hospital setting, which can be attributed to the opening of the PCU in RAM. However, we observed an increase in the percentage of patients who passed away in the emergency service. We posit that despite the opening of the PCU in 2012, the rising demand for PC needs in RAM may have contributed to these findings.

It should be noted that, in 2019, patients with potential indications for palliative care had an average of 1.86 hospitalizations and 3.38 visits to the emergency service in the last 12 months of life. These individuals had a greater number of hospitalizations (p=0.0005) compared to those who did not have potential indications for PC. As hospital admissions in the final months of life are seen as an indicator of inadequate end-of-life care [13], we can infer that further measures may be taken to identify and provide appropriate care to these patients in a timely manner.

We acknowledge the limitations of our study, including the potential for information bias in the electronic records, and the possibility of errors in patient clinical files and the SICO online platform. Additionally, the limited geographic scope of the study to the RAM region, with its unique characteristics as an island, may affect the ability to generalize these results to other areas with greater healthcare support. Further studies, with different designs and populations, are needed to more fully understand the implications of these results.

It is worth noting that the results of this study should be interpreted with caution, as the proportion of individuals with potential indications for palliative care is not the only factor to consider when determining the specific need for such care. Each patient presents a unique set of complexities, and a more comprehensive assessment may be necessary to determine the need for palliative care. Further studies with the purpose of conducting a more detailed evaluation of palliative care needs may be conducted in the future.

## Conclusions

We believe that health services must be adjusted to the intrinsic characteristics and needs of the populations they serve. With this study, we conclude that there has been an improvement in end-of-life care for patients, although we observe an increase in the need for palliative care in RAM. These results provide important information for a possible readjustment of palliative care in RAM.

Finally, the assessment of PC needs in 2019 allows us to have a pre-pandemic overview. We envision a reassessment after two years of the COVID-19 pandemic in Portugal, seeking to identify its possible effects on PC needs in RAM.
